# Transcriptomics combined with physiological analysis provides insights into the mechanism of resistance to *Coleosporium bletiae* in *Bletilla striata*

**DOI:** 10.3389/fpls.2025.1604512

**Published:** 2025-07-14

**Authors:** Qiao Liu, Xi Lu, Qiaofen Wu, Zhibiao Lu, Renjun Qin, Kui Huang, Xun Zou, Ke Xia, Yanni Yang, Shuo Qiu

**Affiliations:** ^1^ Guangxi Institute of Botany, Guangxi Zhuang Autonomous Region and Chinese Academy of Sciences/Guangxi Key Laboratory of Plant Functional Phytochemicals and Sustainable Utilization, Guilin, China; ^2^ Guilin Sanjin Pharmaceutical Co., Ltd., Guilin, China; ^3^ Guangxi Yifang Tianjiang Pharmaceutical Co., Ltd., Guilin, China

**Keywords:** *Bletilla striata*, rust pathogen, transcriptomics, physiological analysis, resistant and susceptible material

## Abstract

**Introduction:**

*Bletilla striata* (Orchidaceae) is a valuable traditional Chinese medicinal plant prized for its dried rhizomes. However, its cultivation faces significant challenges from leaf rust disease caused by *Coleosporium bletiae*, which causes substantial yield losses.

**Methods:**

To investigate host resistance mechanisms, we compared rust-resistant and susceptible *B. striata* accessions through integrated transcriptomic and physiological analyses.

**Results and discussion:**

Phenotypic observations revealed that while both resistant and susceptible plants developed rust spores by 2 days post-inoculation (dpi), the resistant accession exhibited a significantly slower progression of spore stack formation and lesion expansion on abaxial leaf surfaces over time. Integrated transcriptomic and physiological analyses revealed that the rust-resistant material of *B. striata* accessions exhibited faster and stronger defense responses to pathogen infection compared to susceptible plants. These responses were characterized by significant upregulation of DEGs associated with antioxidant defense systems, secondary metabolite biosynthesis, JA, SA, and BR signaling pathways, concurrent downregulation of DEGs involved in cell wall remodeling, and calcium-mediated signaling. Furthermore, rust pathogen inoculation triggered rapid physiological responses in resistant plants, including enhanced activity of defense-related enzymes (CAT, PAL, β-1,3-glucanase, and chitinase) and early accumulation of osmolytes (soluble sugars, soluble proteins, and proline). These coordinated molecular and biochemical responses effectively restricted pathogen colonization and spread. These findings delineate the molecular basis of rust resistance in *B. striata*, identifying key regulatory nodes in defense pathways that could be targeted through precision breeding or genetic engineering to develop durable resistance against *C. bletilla*.

## 1 Introduction

*Bletilla striata* (Thunb.) Reichb.f., a medicinal orchid of the *Bletilla* genus (Orchidaceae), produces polysaccharide-rich dry tubers that have been widely utilized in multiple industries, including traditional Chinese medicine (Jiang et al., 2019; Shao et al., 2024), food, and cosmetics (Chen et al., 2021). Leaf rust, caused by *Coleosporium bletilla*, is one of the most devastating fungal diseases affecting *B. striata*, resulting in significant yield losses (Liu et al., 2023; Xu et al., 2023; Wu et al., 2024). As a member of the *Coleosporium* genus—known to cause rust diseases in multiple medicinal plants—this pathogen severely compromises the growth of *B. striata*, *Tetradium ruticarpum*, *Clematis florida*, and other host species. In China, leaf rust has emerged as a critical constraint to *B. striata* production, particularly in major cultivation regions such as Guizhou, Guangxi, Sichuan, and Hubei provinces (Xu et al., 2023; Liu et al., 2023). Most *Coleosporium* species are macrocyclic autoecious rust fungi, producing multiple spore types that enhance their environmental adaptability (Staples, 2000; Wei et al., 2013; Aime et al., 2018). This complex life cycle, combined with their obligate parasitic nature, makes these pathogens particularly difficult to control. While fungicide application remains a primary control strategy against *C. bletiae* in commercial *B. striata* cultivation, its implementation requires careful consideration of phytotoxicity risks and residue management. Besides that, frequent fungicide applications exert a strong selection pressure on *C. bletiae* populations, driving the evolution of resistant physiological races through. Similar fungicide resistance patterns have been quantitatively characterized in wheat and rice, with 73%–89% efficacy loss occurring within 5 years of continuous treatment (Chen et al., 2014; Hawkins et al., 2019).

In agricultural production, cultivating resistant cultivars remains the primary strategy for disease prevention. However, the strong adaptive capacity and genetic variability of rust pathogens often outpace the development of new resistant varieties. This underscores the critical need to elucidate the host defense mechanisms against *C. bletiae*. Advances in high-throughput sequencing and molecular biology have now made pathogen–plant interaction studies a fundamental approach to develop effective disease control strategies (Gill et al., 2018; Liu et al., 2020)—for example, a recent study on the high-resolution transcriptional profiling of *Phakopsora pachyrhizi* during infection revealed dramatic shifts in metabolic and signal transduction pathways across different stages of the infection cycle, with three apoplastic effectors found to be essential for infection by suppressing the plant immune response (Ouyang et al., 2024). Liu et al. (2022) used the highly susceptible cultivar Chuanyu12 (CY12) to analyze transcriptome profiles following inoculation with *Puccinia striiformis* f. sp. tritici (Pst) and uncovered the regulatory network and molecular mechanisms underlying wheat’s response to Pst infection. These findings demonstrate that global gene expression profiling, particularly through next-generation sequencing (NGS), can elucidate molecular interactions in plant–fungal pathosystems, including non-model host–pathogen systems.

To date, although *C. bletiae* is known to cause severe rust in *B. striata*, the molecular and physiological responses of the host to infection remain poorly understood. This situation severely impedes both the effective control of *B. striata* leaf rust and the sustainable development of the *B. striata* industry. In this study, we utilized resistant and susceptible *B. striata* accessions to investigate physiological and transcriptomic changes following pathogen inoculation. Our findings clarify the defense mechanisms of *B. striata* against *C. bletiae*, providing a theoretical foundation to develop strategies to control this pathogen.

## 2 Materials and methods

### 2.1 Plant material

Based on our research group’s previous resistance evaluation studies (Wu et al., 2024), two materials were selected for this experiment: (1) resistant material “BJ-11”—cited from Honghe, Yunnan Province, China. Plants of this material belong to small tubers and have narrow leaves, pale yellow flowers, and relatively low ornamental value; (2) susceptible material “Guibai 4”—cited from Baoding Mountain, Ziyuan County, Guangxi, China, which has been bred and registered by Guangxi Institute of Botany, Chinese Academy of Sciences (new variety registration no.: Guideng (Medicine) 2015034). Plants of this material have wide leaves, purple flowers, and large tubers, with high ornamental value. To facilitate, these two materials were re-coded as follows: H=resistant, G=susceptible. All plants selected in this study were tissue culture seedlings that were transplanted and acclimated for 3 years.

### 2.2 Methods for morphological identification of rust disease pathogens

The back of the leaf surface is covered with many yellow summer spore piles in powder form (**Supplementary Figure S1**). Observed fresh lesions under a stereomicroscope (×10–×40 magnification) and its key characteristics: bright yellow to orange (due to carotenoid pigments); dry, powdery consistency (indicative of loose spore masses); sorus (spore mass) morphology: pulvinate (cushion-like) with a raised center; non-peridiate (lacking a protective membrane); size: 200–500 um in diameter (visible as discrete pustules) (**Supplementary Figure S2**).

### 2.3 Collection of rust spores and preparation of working fluid

In April, infected *B. striata* leaves exhibiting rust uredinia were collected from experimental plots. The leaves were gently washed under a thin stream of running water, and spores were dislodged from the abaxial surface using a soft toothbrush. The resulting spore suspension was collected in a sterile beaker. The stock suspension was diluted to an optimal concentration of 40–50 spores per visual field at ×100 magnification. To enhance spore adhesion, 0.1% Tween-80 was added to prepare the working inoculum.

### 2.4 Treatment

For inoculation, the spore suspension was evenly sprayed onto the abaxial leaf surfaces of 126 plants representing two experimental materials. Immediately after inoculation, the plants were individually bagged and maintained at high humidity for 24 h. Following this incubation period, the bags were removed, and the plants were spatially separated to prevent cross-contamination.

### 2.5 Sample

Leaf samples (2.0 g) were collected at 0 (pre-inoculation control), 2, 4, and 8 days post-inoculation (dpi), with three biological replicates per time point. For consistency, we selected leaves from identical nodal positions across all plants. Immediately after collection, the midribs and major veins were excised, and the leaf tissue was flash-frozen in liquid nitrogen. All samples were stored at -80°C until subsequent physiological assays and transcriptome sequencing analysis.

### 2.6 Physiological parameter determination

#### 2.6.1 Determination of chlorophyll content

The 95% ethanol grinding–centrifugation method was used to extract the chlorophyll content, and the absorbance of Chl a and Chl b was measured at 665 and 649 nm, respectively (Li et al., 2005).

#### 2.6.2 Determination of antioxidant enzyme activities

##### 2.6.2.1 Superoxide dismutase

Leaf samples (0.5 g fresh weight) were precisely weighed and processed according to the manufacturer’s protocol (Superoxide Dismutase Assay Kit, SOD A001-1; Nanjing Jiancheng Bioengineering Institute, Nanjing, China).

##### 2.6.2.2 Catalase

Leaf samples (0.5 g fresh weight) were precisely weighed and analyzed following the manufacturer’s protocol (Catalase Assay Kit, CAT A007-1-1; Nanjing Jiancheng Bioengineering Institute, Nanjing, China).

##### 2.6.2.3 Phenylalanine ammonia lyase

Leaf samples (0.2 g fresh weight) was precisely weighed and analyzed for phenylalanine ammonia-lyase (PAL) activity following the manufacturer’s protocol (PAL Assay Kit, A137-1-1; Nanjing Jiancheng Bioengineering Institute, Nanjing, China).

#### 2.6.3 Determination of osmotic substances

##### 2.6.3.1 Soluble sugar content

Fresh leaf samples (0.5 g) were precisely weighed and analyzed for soluble sugar content following the manufacturer’s protocol (Soluble Sugar Assay Kit, SS A145-1-1; Nanjing Jiancheng Bioengineering Institute, Nanjing, China).

##### 2.6.3.2 Soluble protein content

The measurement was based on the Coomassie brilliant blue G-250 staining method. The details are as follows: 0.5 g fresh leaves was weighed and fully submerged in liquid nitrogen to grind into a homogenate, and 10 mL of distilled water was added into the homogenate and mixed well to extract at room temperature for 1 h. Then, it was centrifuged at 4,000 rpm for 20 min, and 0.5 mL of the supernatant was taken. Finally, 0.5 mL of distilled water and 5 mL of G-250 solution were added, in turn, mixed well, and left for 2 min. The absorbance value was measured at 595 nm. SP standard curve drawing: A 100-mg sample of bovine serum protein was weighed, and 100 mL water was added as standard protein liquid. It was diluted into different concentrations according to the table (**Supplementary Table S1**). SP (ug/g) = (A*V*D)/W, where A is the corresponding protein content, ug; V is the total volume of extract, 10 mL; Dis the dilution multiple, 2; and W is the fresh weight of the sample (g).

##### 2.6.3.3 Proline content

Proline content was determined with reference to Bates et al. (1973): 0.1 g of leaves was weighed and ground with 3% sulfenicylic acid, extracted in boiling water bath for 10 min, and then centrifuged for 10 min at 10,000 *g*. The reaction system was as follows: 2 mL of supernatant, 2 mL of glacial acetic acid, 2 mL acidic ninhydrin reagent (1.25 g ninhydrin + 30 mL glacial acetic acid + 20 mL 6M phosphoric acid). The reaction was terminated by using an ice bath after 40 min of boiling water bath. The red product was extracted from toluene, and the absorbance at 520 nm was determined.

#### 2.6.4 Determination of major hydrolase activity

Chitinase assay was determined with reference to Boller and Mauch (1988). The substrate preparation was as follows: prepare a 0.5% (w/v) solution of glycol chitin (exhibiting superior solubility compared to colloidal chitin) in 50 mM sodium acetate buffer (pH 5.0). For the enzyme reaction, combine 0.5 mL of substrate solution with 0.5 mL of appropriately diluted enzyme extract in a reaction tube. Incubate the reaction mixture at 37°C for 2 h with gentle agitation. Terminate the enzymatic reaction by adding 0.1 mL of 1 M Na_2_CO_3_. Add sequentially 0.2 mL of 1% (w/v) potassium tetraborate (K_2_B_4_O_7_) and 0.2 mL of DMAB reagent [1% (w/v) p-dimethylaminobenzaldehyde in glacial acetic acid/concentrated hydrochloric acid (9:1, v/v)]. Incubate the mixture at 37°C for 20 min to allow color development. Measure the absorbance at 585 nm against appropriate blank controls.

Generate a standard curve using N-acetylglucosamine (GlcNAc) solutions (0–100 ug/mL) processed identically to experimental samples.

β-1,3-Glucanase activity was measured with reference to Pan et al. (1991). Briefly, the reaction mixture contained 0.5 mL of 0.5% (w/v) laminarin (from *Laminaria digitata*, Sigma-Aldrich) dissolved in 50 mM sodium acetate buffer (pH 5.0) and 0.5 mL of appropriately diluted enzyme extract. After incubation at 37°C for 30 min, the reaction was terminated by adding 1.0 mL of dinitrosalicylic acid (DNS) reagent. The mixture was boiled for 10 min to develop the color, cooled to room temperature, and centrifuged at 10,000 *g* for 5 min. The absorbance of the supernatant was measured at 540 nm against appropriate reagent blanks. The reducing sugars released were quantified using a glucose standard curve (0–100 ug/mL). One unit of enzyme activity was defined as the amount of enzyme required to produce 1 µmol of glucose equivalents per minute under the assay conditions.

### 2.7 RNA-seq sequencing

Samples renamed GCK (control group), G2 (2 dpi), G8 (8 dpi), HCK (control group), H2 (2 dpi), and H8 (8 dpi) were used to extract total RNA, with three biological replicates set up for each treatment. When total RNA was extracted from the sample, the concentration and purity of the raised RNA were tested by using Nanodrop2000, the RNA integrity was determined by agarose gel electrophoresis, and the RQN values were determined by using Agilent5300. Once the requirement is met, the mRNA was enriched by Oligo dT. Then, the mRNA was randomly broken into small fragments of about 300 bp by adding a fragmentation buffer. Under the action of reverse transcriptase, one-strand cDNA was synthesized by mRNA using random primers, and subsequent two-strand synthesis was used to form a stable double-stranded structure. The cDNA was repaired to flatten the ends by adding End Repair Mix, and an A base was then added to the 3 end to facilitate the subsequent addition of the adapter sequence. The products after ligation of the adapter were purified and fragment-sorted, PCR amplified with the sorted products, and purified to obtain the final library. Library fragments were double-end sequenced on the NovaSeq X Plus platform (2×150 bp).

### 2.8 RNA-seq data analysis

Stable quality statistics and quality assessment were performed on the raw data, including the GC content (%), quality distribution statistics, and error rate distribution statistics. High-quality raw data were filtered using software fastx_toolkit (version 0.0.14 http://hannonlab.cshledu/fastx_toolkit), Sickle (https://github.com/najoshi/sickle), and SeqPrep (https://github.com/jstjohn/SeqPrep). Then, the assembly order was optimized and filtered by using Trinity (Version v2.8.5, https://github.com/trinityrnaseq), and the longest transcript served as the reference transcript. Finally, the sample data were compared with the assembled reference to obtain the mapping results for each sample. All transcripts were matched against six major databases (NR, COG, GO, KEGG, Pfam, and Swiss-prot databases) to obtain detailed annotation information. RESM software (version 1.3.1, http://deweylab.biostat.wisc.edu) was used in the quantitative analysis of expression levels, and the standard for measuring expression levels is TPM. Genes with an adjusted *P*-value <0.01 and fold change ≥2 found by DESeq2 were assigned as differentially expressed (version 1.24.0, http://bioconductor.org/package).

### 2.9 Real-time PCR

A total of 10 DEGs, *SOD1* (DN49610), *ETR* (DN531), *ERF* (DN16282), *WRKY33* (DN4172), *MYC2* (DN2177), *XTH* (DN1676), *CPK26* (DN7604), *WRKY22* (DN9292), *KCS* (DN4740), and *CALM* (DN37386), were selected to validate the reliability of transcriptome data by real-time PCR. The primer sequences (**Supplementary Table S2**) were designed by Premier 5. Real-time quantitative PCR experiments was performed with the following system: total 20 uL reaction system consisting of 1 uL cDNA samples,10 uL SYBR Color qPCR Master Mix (2X), 0.4 uL forward primer, 0.4 uL reverse primer, and 7.2 uL ddH_2_O. The PCR cycling experiments were performed using an ABI7300 fluorescent quantitative with the cycling conditions: 95°C–5 s, 72°C–40 s, 60°C–30 s, 40 cycles.

### 2.10 Data statistics and analysis

The data were collated using Microsoft Excel 2019 software, and the one-way analysis of variance (one-way ANOVA) statistical analysis was performed with SPSS26 software after normal test of data (Tukey’s HSD test, α = 0.05). All data were set in three replicates, and the mean value ± standard error was taken. Graphs were drawn by using GraphPad prism 9.3, and heatmaps and network graph were plotted by R studio (version 2.4.3) and Cytoscape (version 3.10.2).

## 3 Results

### 3.1 Morphological response of susceptible and resistant materials to the inoculated rust pathogen

**Figure 1:**
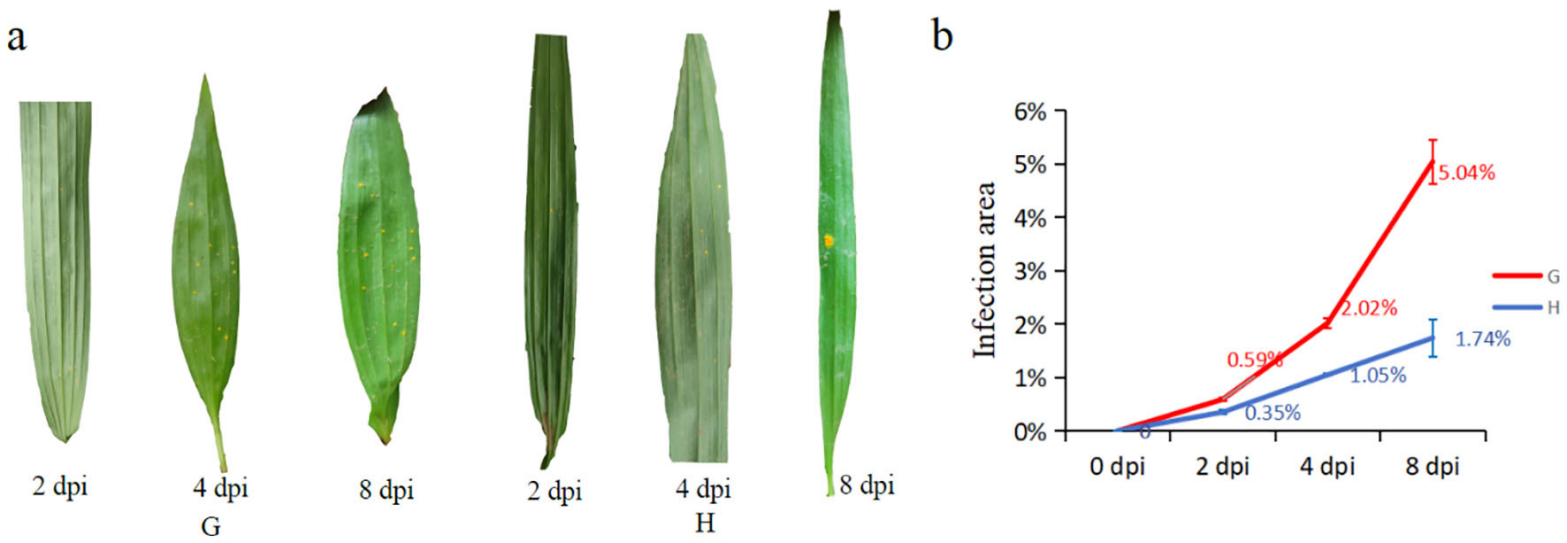
Infection process of susceptible (G) and high-resistance (H) materials after inoculation of rust pathogen: **(a)** phenotypic changes of leaves after inoculation; **(b)** trends of leaves’ infected area between two materials of different days post-inoculation (dpi).

The results showed that orange rust fungi (*C. bletiae*) could be easily observed on the abaxial surface of leaves in both susceptible and resistant materials of *B. striata* as early as the 2nd dpi (**Figure 1a**). However, in the susceptible material, the number of urediniospore (summer spore) stacks increased significantly, and the infection area expanded rapidly over time (**Figure 1b**). In contrast, the high-resistance material exhibited minimal increases in urediniospore production and infection area until 8 dpi. These findings demonstrated that resistant material possessed partial resistance to rust pathogens, effectively slowing the progression of infection compared to susceptible material.

### 3.2 Physiology response of susceptible and resistant materials to the inoculated rust pathogen

To investigate the disease resistance mechanisms, we examined the physiological responses of leaves from both plant materials during pathogen infection.

#### 3.2.1 Chlorophyll content

**Figure 2:**
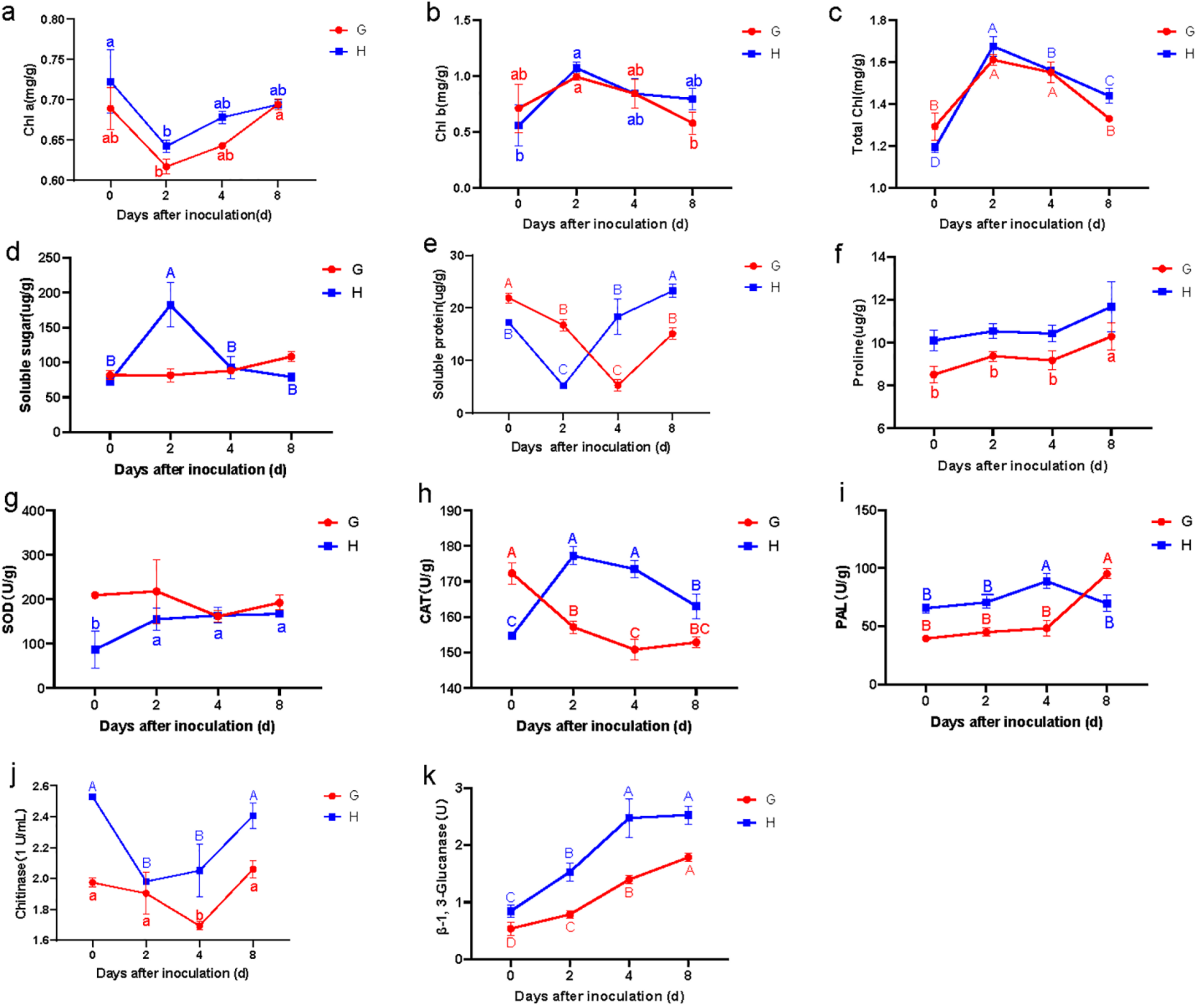
Changes of leaf physiological parameters in susceptible (G) and resistant (H) materials on different days after inoculation: Chl a **(a)**, Chl b **(b)**, Total chl(total chlorophyll) **(c)**, Soluble sugar **(d)**, Soluble protein **(e)**, Proline **(f)**, SOD **(g)**, CAT **(h)**, PAL **(i)**, chitinase **(j)**, and β-1,3-glucanase **(k)**. Lowercase letters (a, b, c) represent the differences at *p* < 0.05.

Following pathogen inoculation, the chlorophyll content (Chl a, Chl b, and total chlorophyll) in both resistant and susceptible materials of *B. striata* exhibited similar temporal trends, though their resistance levels differed (**Figures 2a–c**). Specifically, the Chl a levels initially decreased before recovering after 2 dpi. Chl b and total chlorophyll showed an inverse pattern, rising transiently before declining at 2 dpi. This synchronized inflection at 2 dpi implied that rust infection reprogramed the chlorophyll metabolism in both genotypes, possibly due to pathogen-induced oxidative stress damaging the photosynthetic apparatus (Kariola et al., 2005), or remobilization of chlorophyll-derived nutrients for defense (Hörtensteiner and Kräutler, 2011). Notably, the parallel response in susceptible and resistant plants suggested that chlorophyll dynamics alone did not determine resistance outcomes.

#### 3.2.2 Soluble sugar, soluble protein, and proline content

Compared to the susceptible material, the resistant material exhibited a transient but significant increase in leaf soluble sugar content, peaking during early infection before returning to baseline levels (**Figure 2d**). This pattern aligns with findings that sugars act as signaling molecules and energy sources for defense responses (Bolouri Moghaddam and Van den Ende, 2012; Trouvelot et al., 2014). In contrast, the susceptible material showed no notable change, suggesting failure in sugar-mediated defense activation.

Both materials displayed a similar trend in soluble protein content, characterized by an initial decrease (likely due to pathogen-induced hydrolysis) followed by recovery. However, the timing of the nadir differed: the resistant material reached its minimum at 2 dpi, while the susceptible material lagged, reaching its lowest point at 4 dpi (**Figure 2e**). This kinetic difference implies that the resistant material reprograms its metabolism more rapidly, possibly to prioritize defense-related protein synthesis (PR proteins and enzymes for phytoalexin production).

The proline content followed nearly identical trends in both materials, with no significant differences observed in resistant material, but significantly increasing at 8 dpi in susceptible material (**Figure 2f**). Interestingly, the proline content in the resistant material consistently exhibited higher levels compared to the susceptible counterpart, suggesting a pivotal role for proline in basal resistance against pathogen infection. Proline may function as both an osmoprotectant and a reactive oxygen species (ROS) scavenger, potentially contributing to enhanced cellular homeostasis during biotic stress (Hayat et al., 2012; Chen et al., 2018). These synergistic functions may collectively enhance the plant’s capacity to restrict pathogen colonization and mitigate biotic stress.

### 3.3 Superoxide dismutase, catalase, and phenylalaninammo-nialyase activities

The SOD activity in resistant material exhibited a distinct pattern, increasing significantly during the first 2 dpi before stabilizing (**Figure 2g**). In contrast, the susceptible material showed a marked decrease in SOD activity between 2 and 4 dpi. This differing response aligns with previous studies demonstrating that SOD plays a crucial role in mitigating oxidative stress during a pathogen attack, suggesting that SOD activity may serve as a key physiological marker for resistance to rust.

The CAT activity demonstrated even more pronounced differences between resistant and susceptible materials. The resistant material displayed a rapid increase in CAT activity, peaking at 2 dpi before gradually declining, though remaining significantly elevated compared to the susceptible material throughout the observation period (**Figure 2h**). Conversely, the susceptible leaves showed declining CAT activity, with the most dramatic reduction occurring within the first 2 dpi. These results strongly indicated that the observed differences in CAT activity likely contributed substantially to the contrasting resistance phenotypes in *B. striata*. The sustained CAT activity was critical to detoxify hydrogen peroxide (H_2_O_2_) and prevent oxidative damage in resistant plants.

Both materials exhibited similar temporal patterns in phenylalanine ammonia-lyase (PAL) activity during the initial 4 dpi, though absolute levels were consistently higher in the resistant material (**Figure 2i**). This elevated baseline PAL activity likely enhanced the resistant material’s defensive capacity, as PAL is a key enzyme in the phenylpropanoid pathway, which produces antimicrobial phytoalexins and lignin. Interestingly, beyond 4 dpi, the susceptible material showed a higher PAL activity than the resistant material, suggesting that PAL may function differently in late-stage infection responses compared to early defense mechanisms. This observation is consistent with studies indicating that pathogen susceptibility can sometimes trigger delayed or dysregulated PAL induction (Mauch-Mani and Slusarenko, 1996).

### 3.4 Chitinase and β-1,3-glucanase activity

The chitinase activity in resistant material was constitutively higher than in susceptible material prior to inoculation. Following pathogen infection, the resistant material exhibited a rapid decrease in chitinase activity, reaching its nadir at 2 dpi before rebounding sharply (**Figure 2j**). In contrast, the susceptible material showed only a gradual decline in chitinase activity during 0–4 dpi, followed by a delayed increase. These results demonstrated that while both materials displayed similar response patterns, the resistant material responded more rapidly and intensely to pathogen infection.

For β-1,3-glucanase activity, both materials showed continuous elevation after inoculation, but the increase was significantly more pronounced in the resistant material (**Figure 2k**). Collectively, these findings indicated that although both chitinase and β-1,3-glucanase activities responded similarly to pathogen infection in both materials, the resistant material exhibited superior sensitivity, faster response kinetics, and greater enzymatic induction. This heightened and more rapid activation of pathogenesis-related protein (PR) appeared to be a crucial component of the enhanced rust resistance observed in the resistant material of *B. striata*.

### 3.5 Principal component analysis

**Figure 3:**
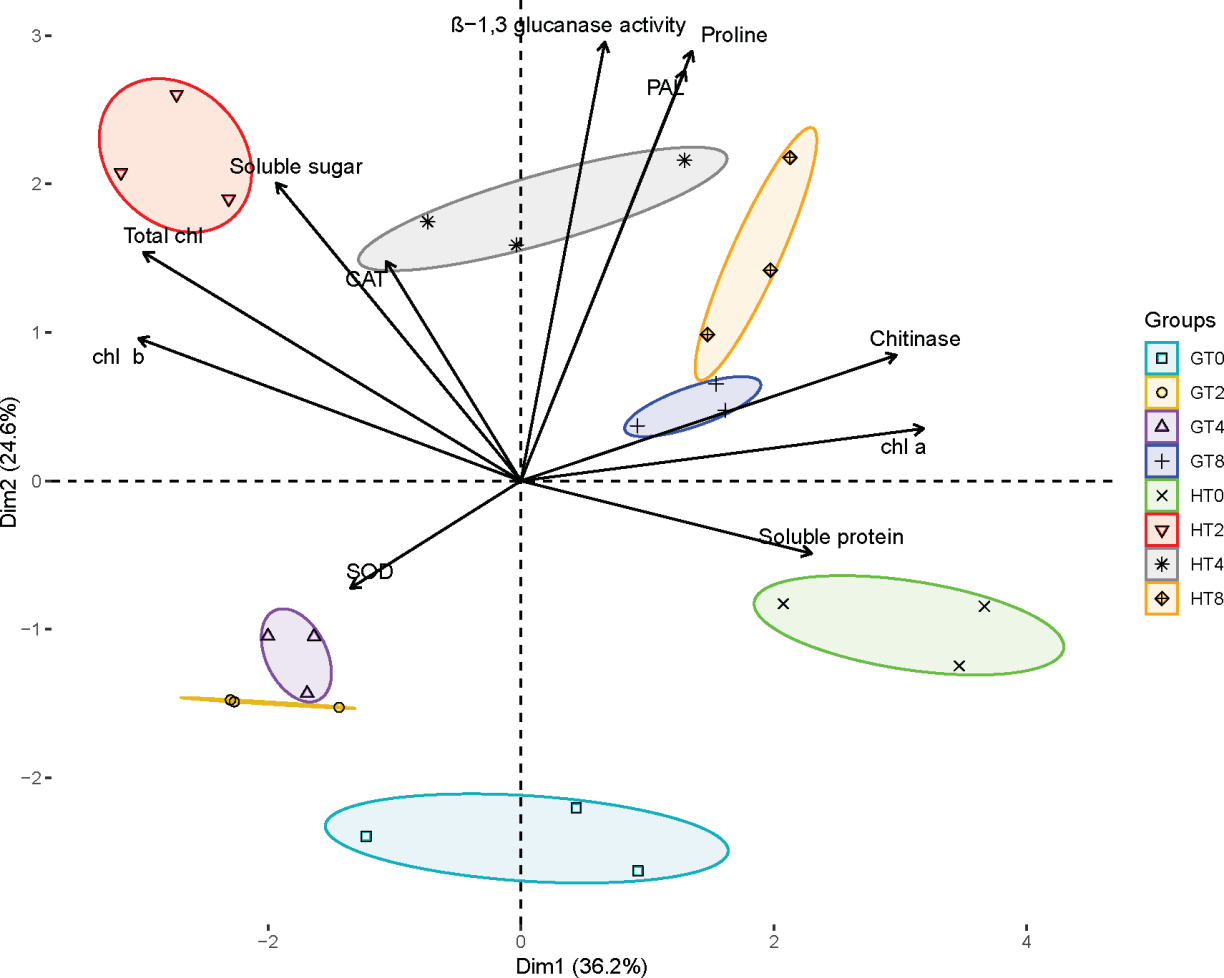
Principal component analysis plot. Effect of pathogen infection on various biochemical parameters in plant. Different groups are indicated by different colors and symbols: GT0, GT2, GT4 and GT8 represent 0, 2, 4, and 8 dpi of susceptible material in *B. striata*. HT0, HT2, HT4, and HT8 represent the dpi of resistant material in *B. striata.* Arrows represent the contribution of each variable, with the direction and length indicating their influence on the principal components.

To elucidate the pathogen resistance mechanisms in the resistant material of *B. striata*, we conducted principal component analysis (PCA) to evaluate the contributions of various physiological indicators (**Figure 3**). The PCA revealed a clear separation between susceptible (GT0, GT2, GT4, and GT8) and resistant (HT0, HT2, HT4, and HT8) materials along the two principal components. PC1 (36.2% of variance) reflected differences in basal physiological states, represented by chlorophyll content, chitinase activity, soluble proteins, and soluble sugars. PC2 (24.6% of variance) primarily captured variations in the antioxidant defense system, including PAL, β-1,3-glucanase, proline, SOD, and CAT activities. Notably, while the GT0 and HT0 groups clustered similarly along PC1 (consistent with their pre-inoculation status), HT0 was distinctly separated from GT0 along PC2. This separation associated HT0 with higher baseline levels of Chl a, chitinase, and soluble proteins, suggesting constitutive physiological advantages that may contribute to innate pathogen resistance in the resistant material. Following pathogen infection, the materials exhibited divergent response patterns: at 2 dpi, HT2 clustered with soluble sugars, total chlorophyll, Chl b, and CAT, while GT2 remained associated primarily with SOD activity. By 4 dpi, HT4 showed a strong coordination with β-1,3-glucanase, CAT, PAL, proline, and soluble sugars, whereas GT4 maintained its SOD-associated profile, indicating delayed antioxidant system activation in the susceptible material. At 8 dpi, while both materials showed some convergence in their associations with defense-related parameters (β-1,3-glucanase, CAT, PAL, proline, chitinase, Chl a, and soluble proteins), the HT8 group exhibited a markedly stronger expression of these traits based on its position along both PC axes. These findings demonstrated that the resistant material mounted a faster and more robust defense response through the rapid activation of the antioxidant system (PAL, CAT) and PR (β-1,3-glucanase, chitinase), enhanced physiological maintenance (chlorophyll preservation, osmotic regulation), and earlier metabolic reprogramming (soluble sugar accumulation). The temporal progression of these responses highlighted a coordinated defense strategy in the resistant material of *B. striata* that was both more rapid and more intense than in susceptible plants, ultimately contributing to its superior rust resistance.

### 3.6 Transcriptome sequencing analysis

#### 3.6.1 Results of RNA sequencing

To comprehensively characterize the transcriptional dynamics underlying *Bletilla striata*’s response to pathogen infection, we performed an RNA-seq analysis of resistant (H) and susceptible (G) materials at three critical time points (0, 2, and 8 dpi). The RNA library consisting of 18 samples including resistant and susceptible materials was completed (**Supplementary Table S3**). High-quality sequencing generated 121.64 Gb clean data, evenly distributed between resistant and susceptible materials. *De novo* assembly produced 132,376 unigenes, with mapping rates of >76% validating data and assembly reliability (**Supplementary Table S4**).

#### 3.6.2 Differentially expression genes and its KEGG pathway enrichment analysis

**Figure 4:**
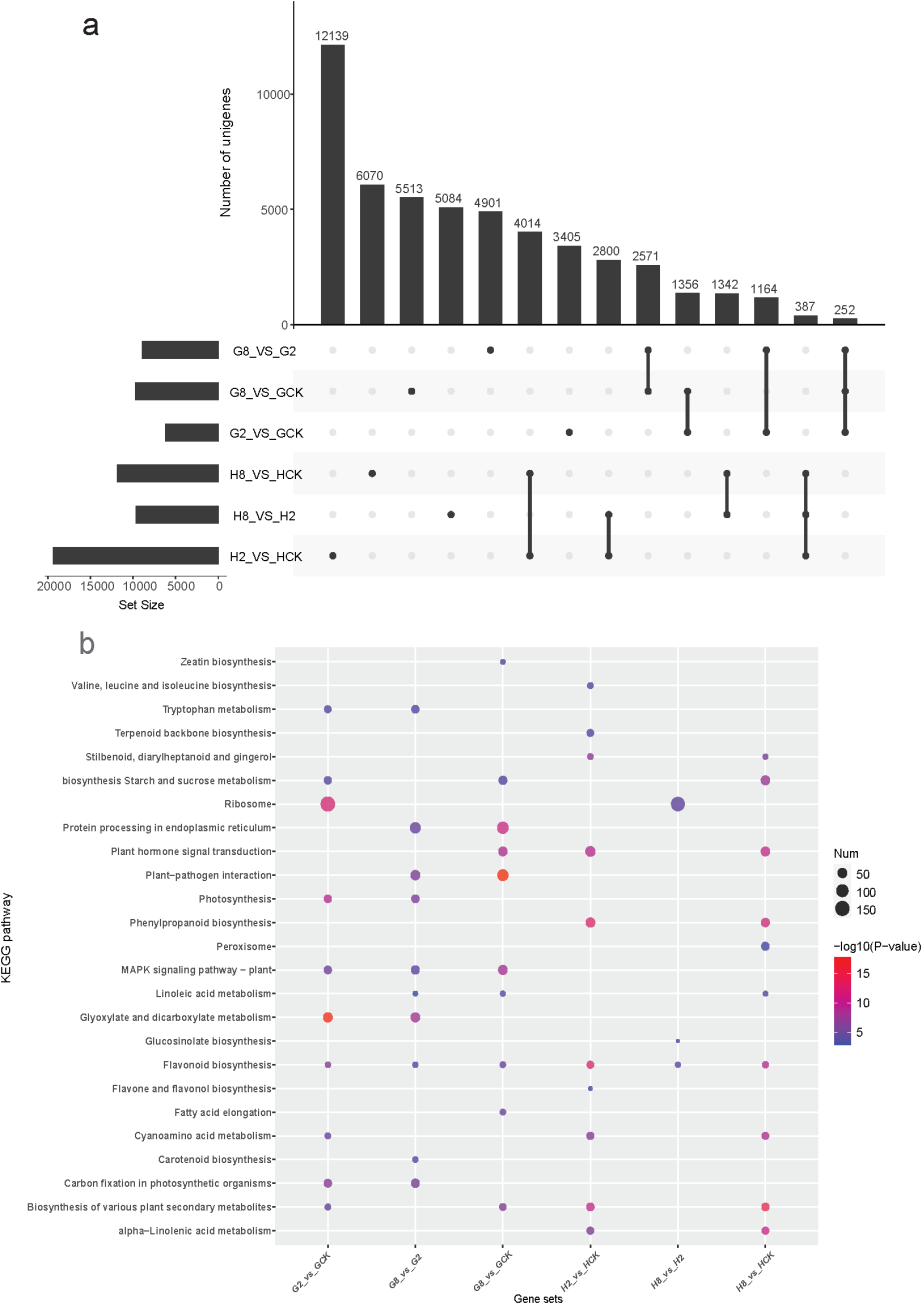
**(a)** Upset showing the shared and unique DEGs among different groups. **(b)** Bubble diagram of the top 10 KEGG pathway enriched by DEGs in different gene sets. GCK, G2, and G8 represent samples on 0, 2, and 8 dpi in susceptible material. HCK, H2, and H8 represent samples on 0, 2, and 8 dpi in resistant material.

Comparative transcriptomic analysis revealed distinct patterns of pathogen-responsive gene expression between resistant and susceptible materials. The resistant material exhibited significantly greater numbers of DEGs between all examined time points (HCK vs. H2, HCK vs. H8, and H2 vs. H8 days after inoculation) compared to the susceptible material (**Supplementary Figure S3**). This pronounced transcriptional response suggested that the resistant material activated a more extensive defense-related gene network upon pathogen infection. The transcriptomic analysis revealed the specific timing of maximal defense activation in the different materials. The resistant material exhibited its strongest response at 2 dpi, with 12,139 DEGs identified in the H2_vs_HCK comparison. In contrast, the susceptible material showed delayed activation, reaching a peak response (5,513 DEGs) only at 8 dpi (**Figure 4a**). This temporal disparity in transcriptional reprogramming suggested that the resistant material mounted an earlier and more intensive defense response at the 2 dpi time point, while the susceptible material showed both delayed and attenuated gene expression changes.

To elucidate the functional characteristics of DEGs, we conducted a KEGG pathway enrichment analysis on each comparison group (**Figure 4b**). The results revealed substantial divergence in the molecular response pathways between the resistant and susceptible materials of *B. striata* after pathogen inoculation. KEGG analysis revealed both shared and material-specific pathway responses to pathogen infection in *B. striata*. Three metabolic pathways were consistently activated in both resistant and susceptible materials: flavonoid biosynthesis (ko00941), cyanoamino acid metabolism (ko00460), and biosynthesis of various plant secondary metabolites (ko00999). The conserved induction of these pathways suggested that they represented core biochemical responses to pathogen infection in *B. striata*, potentially contributing to antimicrobial compound production (flavonoids), detoxification mechanisms (cyanoamino acid metabolism), and broad-spectrum defense compound synthesis (secondary metabolites). However, the susceptible material showed an exclusive activation of several fundamental metabolic pathways: zeatin biosynthesis (ko00908), carotenoid biosynthesis (ko00906), tryptophan metabolism (ko00380), glyoxylate and dicarboxylate metabolism (ko00630), fatty acid elongation (ko00062), protein processing in endoplasmic reticulum (ko04141), and MAPK signaling pathway-plant (ko04016). In contrast, the resistant material specifically activated defense-oriented pathways: phenylpropanoid biosynthesis (ko00940), stilbenoid/diarylheptanoid biosynthesis (ko00945), glucosinolate biosynthesis (ko00966), α-linolenic acid metabolism (ko00592), terpenoid backbone biosynthesis (ko00900), valine/leucine/isoleucine biosynthesis (ko00290), and peroxisome pathway (ko04146). This stark contrast in pathway activation suggested that the susceptible plants maintained basic metabolism while mounting limited defense, while the resistant plants prioritized defense compound production and oxidative stress management.

In respect to the response time to pathogen, the susceptible material primarily activated fundamental metabolic processes during the initial infection stage (2 dpi). The top 10 enriched KEGG pathways included primary metabolic pathways (ko00500, ko00630, ko00710, and ko03010) stress-responsive pathways (ko04016 and ko00380), and secondary metabolic pathways (ko00941, ko00460, and ko00999). Notably, the photosynthesis-related pathways (ko00195) remained significantly enriched, suggesting that the susceptible plants prioritized maintaining basic metabolic functions rather than activating specialized defense mechanisms during early infection. Nevertheless, the resistant material of *B. striata* activated a comprehensive defense-related metabolic network as early as 2 dpi. The enriched pathways were predominantly associated with specialized metabolite biosynthesis and defense signaling, such as amino acid-derived defenses (ko00290 and ko00460), phenylpropanoid pathway activation (ko00940, ko00941, ko00944, and ko00945), terpenoid and lipid mediators (ko00900 and ko00592), defense signaling systems (ko04075), and broad metabolic networks (ko00999). At the peak infection stage (8 dpi) of the susceptible material, it eventually activated partial defense pathways that were already induced in resistant plants at 2 dpi, especially plant hormone signal transduction (ko04075). This demonstrated that while both genotypes possess a similar defense machinery, their differing timing of pathway activation critically determines resistance outcomes. This delayed response coincided with visible disease symptom development.

#### 3.6.3 Time-course analysis of DEGs

To investigate the expression dynamics of DEGs, a time-course analysis was conducted by clustering genes separately for each material.

**Figure 5:**
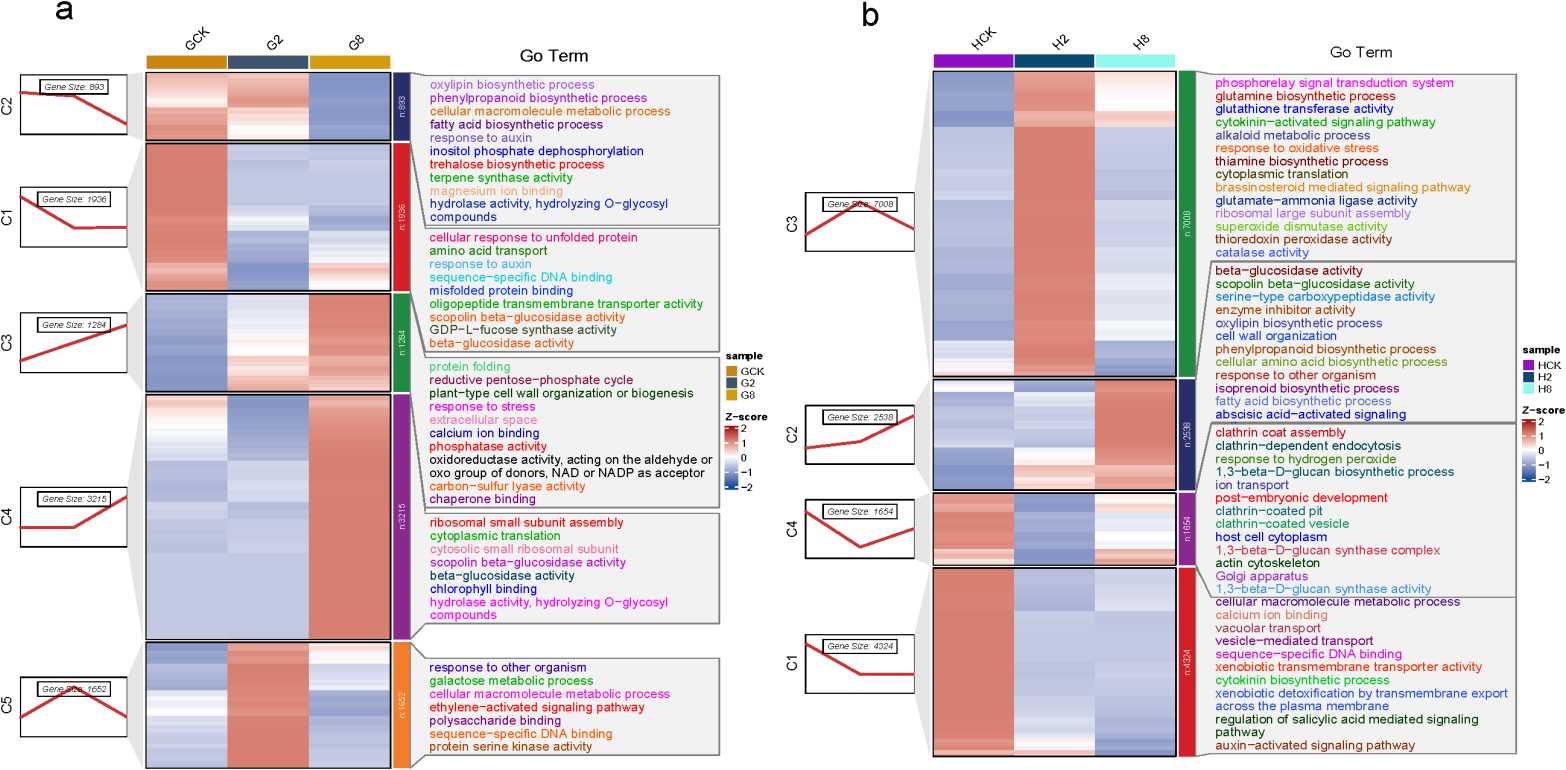
Time-course analysis of dynamic gene expression changes after pathogen inoculation in the different materials of *B. striata*: **(a)** GCK, G2, and G8 represent samples on 0, 2, and 8 dpi in susceptible material; **(b)** HCK, H2, and H8 represent samples on 0, 2, and 8 dpi in resistant material.

The results showed five clusters that were divided from 8,980 DEGs in the susceptible material, which included 1,936 (C1), 893 (C2), 1,284 (C3), 3,215 (C4), and 1,652 (C5) genes, respectively (**Figure 5a**). The 1,936 genes in cluster C1 exhibited a significant downregulation within 2 dpi, maintaining a suppressed expression through 8 dpi. GO enrichment revealed that these genes were predominantly associated with protein quality control systems (cellular response to unfolded protein GO:0006986, misfolded protein binding GO:0051787), metabolic transport processes (amino acid transport GO:0006865, oligopeptide transmembrane transporter activity GO:0008525), phytohormone and defense regulation (response to auxin GO:0009733, sequence-specific DNA binding GO:0043565), and specialized metabolic enzymes (scopolin β-glucosidase activity GO:0102250, β-glucosidase activity GO:000842, GDP-L-fucose synthase activity GO:0047213). The rapid suppression suggested pathogen-mediated disruption of protein homeostasis systems, inhibition of defense-related enzymatic activities, and manipulation of auxin signaling pathways. The sustained downregulation indicated the successful pathogen establishment, long-term suppression of host surveillance systems, and potential suppression of glucosidase-mediated defense compound activation. The 893 genes in cluster C2 exhibited a distinct expression pattern, which maintained high basal expression until 2 dpi, underwent rapid downregulation by 2 dpi, and sustained low expression through 8 dpi. GO enrichment revealed that these genes were primarily involved in defense-related biosynthetic pathways (oxylipin biosynthetic process GO:0031408, phenylpropanoid biosynthetic process GO:0009699, and terpene synthase activity GO:0010333), metabolic regulation (fatty acid biosynthetic process GO:0006633, trehalose biosynthetic process GO:0005992, and inositol phosphate dephosphorylation GO:0046856), and signaling and enzymatic functions (response to auxin GO:0009733, hydrolase activity GO:0016798, hydrolyzing O-glycosyl compounds GO:0004553, and magnesium ion binding GO:0000287). The initial high expression suggested that its basal defense mechanisms were constitutively active pre-infection, while the subsequent suppression indicated successful pathogen interference with defense biosynthesis and targeted disruption of key metabolic pathways, like JA signaling precursors (oxylipins), antimicrobial compound production (phenylpropanoids and terpenes), and stress-protectant synthesis (trehalose). The 1,284 genes in cluster C3 exhibited rapid upregulation following pathogen inoculation, demonstrating the plant’s acute stress response. Functional enrichment analysis revealed that these genes were primarily associated with oxidative stress management [response to stress GO:0006950, oxidoreductase activity (acting on NAD/NADP) GO:0016620, and calcium ion binding GO:0005509], protein homeostasis (protein folding GO:0006457 and chaperone binding GO:0051087), metabolic reprogramming (reductive pentose-phosphate cycle GO:0019253 and carbon-sulfur lyase activity GO:0016844), structural defenses (plant-type cell wall organization GO:0009664 and extracellular space localization GO:0005615), and signaling components (phosphatase activity GO:0016791). The immediate upregulation indicated the successful pathogen recognition, rapid deployment of general defense mechanisms, and activation of both local and systemic responses. These key functional implications included enhanced ROS scavenging capacity, increased protein quality control (chaperones and folding), metabolic shifts toward defense (pentose-phosphate pathway), and structural reinforcement (cell wall reorganization). The 3,215 genes in cluster C4 exhibited a distinct temporal pattern, which maintained a low basal expression until 8 dpi and underwent significant upregulation at 8 dpi. Functional enrichment revealed that these late-induced genes were primarily involved in protein synthesis (ribosomal small subunit assembly GO:0000028, cytoplasmic translation GO:0002181, and cytosolic small ribosomal subunit GO:0022627), specialized metabolic enzymes (scopolin β-glucosidase activity GO:0102250, β-glucosidase activity GO:0008422, hydrolase activity GO:0016798, and hydrolyzing O-glycosyl compounds GO:0004553), and photosynthetic components (chlorophyll binding GO:0016168). This late-stage induction suggested the pathogen manipulation of host cellular processes, establishment of compatible interaction, and potential nutrient redirection to support pathogen growth. The 1,652 genes in cluster C5 displayed immediate upregulation at 2 dpi and subsequent downregulation through 8 dpi. These transiently activated genes were associated with defense response pathways (response to other organism GO:0051707 and ethylene-activated signaling pathway GO:0009873), metabolic processes (galactose metabolic process GO:0006012 and cellular macromolecule metabolic process GO:0044260), and molecular functions (protein serine kinase activity GO:0106310, sequence-specific DNA binding GO:0043565, and polysaccharide binding GO:0030247). The result indicated early pathogen recognition and signaling rapid suppression with advancing infection. Ethylene signaling likely mediated early defense, and metabolic shifts may have supported initial resistance. The subsequent suppression reflected pathogen interference with defense signaling.

A total of 15,569 DEGs were divided into four clusters (C1, 4,324; C2, 2,538; C3, 7,008; and C4, 1,654) based on their expression trend in the resistant material (**Figure 5b**). The resistant material showed rapid downregulation by 2 dpi and sustained through 8 dpi. The functional enrichment revealed several key pathways, including membrane transport processes (vacuolar transport GO:0007034, vesicle-mediated transport GO:0016192, xenobiotic transmembrane transporter activity GO:0008529, and transmembrane export GO:0140359), hormone signaling networks (cytokinin biosynthetic process GO:0009691, auxin-activated signaling GO:0009734, and salicylic acid signaling regulation GO:2000032), and molecular recognition systems (calcium ion binding GO:0005509 and sequence-specific DNA binding GO:0043565). The coordinated downregulation may suggest the strategic suppression of a specific recognition machine, potential pathogen avoidance mechanisms, and reprogramming of hormone signaling networks. It also showed rapid pathogen recognition by calcium signaling and DNA-binding proteins and defense prioritization though SA/auxin/cytokinin modulation. The resistant material exhibited a distinct activation pattern in cluster C2 upon pathogen inoculation and then, significantly, upregulation since 2 dpi. Functional enrichment revealed that these genes were predominantly associated with defense-related enzymatic activities (β-glucosidase activity GO:0008422, scopolin β-glucosidase activity GO:0102250, serine-type carboxypeptidase activity GO:0004185, and enzyme inhibitor activity GO:0004857), specialized metabolite biosynthesis (phenylpropanoid biosynthetic process GO:0009699, oxylipin biosynthetic process GO:0031408, isoprenoid biosynthetic process GO:0008299, and fatty acid biosynthetic process GO:0006633), structural defenses (cell wall organization GO:0071555), and signaling pathways (abscisic acid-activated signaling GO:0009738 and response to other organisms GO:0051707). The result suggested that a secondary defense layer may be activated with pathogen life cycle transitions, which potentially prevented late-stage pathogen establishment. It might consist of β-glucosidase-activated defense compounds from glycosylated precursors, antimicrobial metabolites produced by phenylpropanoid/oxylipin pathways, cell wall reinforcement to limit pathogen spread, and stress responses mediated through ABA signaling. The largest gene cluster C3 (*n* = 3,215 genes) exhibited a characteristic response pattern, with significant upregulation from 0 to 2 dpi and downregulation since then, represented as the most dramatic transcriptional shift in the resistant material. Functional enrichment revealed two core defense systems, namely, oxidative stress management (response to oxidative stress GO:0006979, superoxide dismutase activity GO:0004784, catalase activity GO:0004097, and thioredoxin peroxidase activity GO:0008379) and hormonal signaling networks (cytokinin-activated signaling GO:0009736 and brassinosteroid- mediated signaling GO:0009742). It showed that coordinated defense activation consisted of synchronized antioxidant and hormonal responses, resulting in early ROS management to prevent cellular damage and defense resource allocation through hormonal crosstalk. The genes in cluster C4 exhibited significant downregulation during 0–2 dpi, but subsequent upregulation after that. Three core cellular processes were enriched, including membrane trafficking systems (clathrin coat assembly GO:0048268, clathrin-dependent endocytosis GO:0072583, clathrin-coated vesicle formation GO:0030136, and golgi apparatus organization GO:0007030), cell wall reinforcement (1,3-β-D-glucan biosynthetic process GO:0051274, 1,3-β-D-glucan synthase activity GO:0003843, and glucan synthase complex formation GO:0000148), and stress response mechanisms (response to hydrogen peroxide GO:0042542, actin cytoskeleton reorganization GO:0030036, and ion transport regulation GO:0006811). DEGs that were downregulated during the early phase (0–2 dpi) may represent the temporary suppression of constitutive endocytosis, which may contribute to pattern recognition receptor stabilization and pathogen-associated molecular pattern (PAMP) recognition. Then, they were upregulated in the late phase (2–8 dpi), implying the activation of callose deposition (1,3-β-glucan synthesis), membrane repair mechanism, and cellular compartmentalization.

#### 3.6.4 Key genes analysis

To elucidate the molecular mechanisms underlying pathogen resistance in *B. striata*, we conducted analyses of DEGs associated with five critical defense-related functional categories, including cell wall reinforcement systems, phytohormone signaling networks, plant–pathogen interaction, oxidative stress management, and antimicrobial biosynthesis pathways.

**Figure 6:**
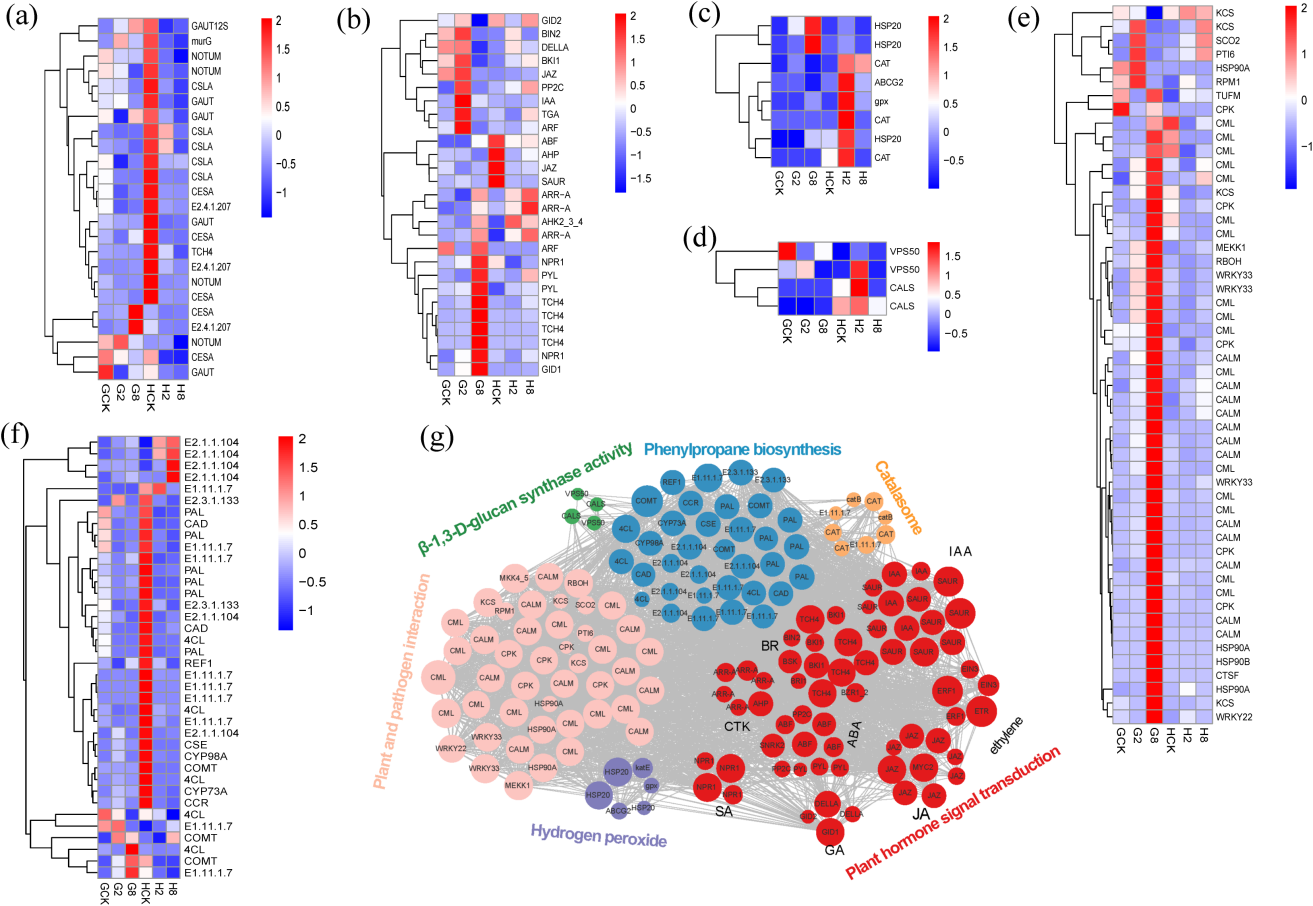
Heatmap of genes from cell wall organization **(a)**, plant hormone signal transduction **(b)**, response to oxidative stress **(c)**, β-1,3-D-glucan synthase activity **(d)**, plant and pathogen interaction **(e)**, and phenylpropane biosynthesis **(f)**. GCK, G2, and G8 represent samples on 0, 2, and 8 dpi in susceptible material; HCK, H2, and H8 represent samples on 0, 2, and 8 dpi in resistant material. Gene co-expression regulatory networks of response to rust in *B. striata* **(g)**. The colored nodes denote genes involved in different pathways, the edges stand for interactions between genes, and the gray lines prove interactions between genes.

The results showed that genes related to the synthesis of cell wall components pectinase and cellulase (*GAUT*, *CSLA*, *NOTUM*, *CESA*, and *TCH4*) were significantly downregulated after inoculation in resistant plants compared to susceptible plants. It indicated that these genes were involved in the rapid response to pathogen, and they provided the resistant plants with resistance of rust by reducing cell wall plasticity to enhance structural integrity against rust pathogen (**Figure 6a**).

Many genes involved in the pathway of plant hormone signal transduction, such as *IAA*, *ARF*, *JAZ*, *TGA*, *ABF*, *ARR-A*, *AHP*, *NPR1*, *GID1*, and so on, showed different ways of expression in the different materials, which indicated that hormonal signal varied in response to the pathogen between the different materials (**Figure 6b**). Within the auxin signaling pathway, *IAA* (a negative regulator) and *ARF* transcriptional activators were significantly upregulated at 2 dpi in the susceptible material, whereas *SAUR* (a growth-promoting effector) was strongly downregulated at 2 dpi in the resistant material, indicating opposing hormonal strategies in rust pathogen defense. This inverse regulation implied that auxin signaling bifurcates defense strategies: promoting susceptibility (*IAA*/*ARF*↑) or resistance (*SAUR*↓). At the same times, the JA, ABA, and SA f signal pathways were activated in the resistant material by the downregulation of *JAZ*, *ABF*, and *TGA*, respectively, at 2 dpi, while the JA and ABA signal pathways were depressed in the susceptible material by the upregulation of *JAZ* and *PP2C*. Notably, two critical BR signaling regulators showed coordinated upregulation at 2 dpi in the susceptible material: *BIN2* (GSK3 kinase) and its interacting partner *BKI1*. This dual induction suggested a strategic BR signaling defense–growth integrator during early infection. Besides that, downregulation *AHP* and *GID2* in the resistant material at 2 dpi showed that cytokinin signaling potentiation and growth signal suppression were mediated by GA. All of the results above showed that early suppression of growth-related signals (auxin, cytokinin, and GA) and concurrent activation of defense hormones (SA, JA, ABA, and BR) in the resistant material provided the evidence of precise hormonal rebalancing with growth–defense tradeoff initiation and prioritization of defense signaling, resulting in earlier response timing than susceptible plants.

The resistant plants exhibited a rapid, coordinated activation of antioxidant defenses post-inoculation, contrasting sharply with susceptible counterparts. The key components were significantly upregulated, involving ROS-scavenging enzymes: two catalases (*CAT*), glutathione peroxidase (*gpx*), stress protectants: heat shock protein gene (*HSP20*), and detoxification transporters: ATP-binding cassette transporter (*ABCG2*) (**Figure 6c**). This transcriptional reprogramming correlated with the accelerated accumulation of SOD and CAT, demonstrating a three-tiered ROS management strategy that consists of enzymatic detoxification, protein protection, and toxin efflux. The sustained oxidative burst control likely contributes to the resistant plants’ ability to maintain redox homeostasis during a pathogen attack.

In addition, our analysis revealed distinct expression patterns of key callose-related genes between resistant and susceptible materials of *B. striata*. We focused on genes associated with β-1,3-D-glucan synthase activity and found that *VPS50* and *CALS* genes responded differently to pathogen in the different materials. These genes were significantly upregulated at 2 dpi, associated with chitin synthase as well as callose synthesis, probably resulting in improving resistance to the rust pathogen through targeted vesicle transport of callose synthases, plasma membrane localization and efficient papilla formation (**Figure 6d**). β-1,3-D-glucan was an important component of the fungal cell wall (Champer et al., 2016), and this gene was involved in the structural construction and functional regulation of the cell wall and related to disease resistance.

Simultaneously, multi-layered defense reprogramming was observed in resistant plants, involving coordinated regulation of early signaling systems, calcium sensors (*CALM* and *CPK*), MAPK cascade (*MEKK1* and *MKS1*), ROS burst machinery (*RBOH* and *OXI1*), energy homeostasis (*ATP* synthase, *NME*), ROS scavenging (*CAT*), transcriptional control *WRKY22* activation (defense promotion), and *WRKY33* suppression (resistance-associated) (**Figure 6e**). Notably, *WRKY33* showed genotype-dependent regulation, upregulated in susceptible but downregulated in resistant material, suggesting its role as a susceptibility factor when over-expressed and defense-balancing regulator when suppressed. This aligns with the reports on *WRKY33*’s dual role in the SA/JA cross-talk (Birkenbihl et al., 2017).

Genes involved in phenylpropanoid biosynthesis, a central pathway for producing diverse secondary metabolites, were also analyzed. The results revealed that multiple lignin synthesis-related genes (*PAL*, *4CL*, *CAD*, *CYP98A*, *COMT*, and *CYP73A*) were significantly downregulated in resistant material. This suggests that enhanced disease resistance may be achieved through the modulation of lignin biosynthesis, thereby regulating cell wall fortification following rust pathogen inoculation. Additionally, two methyltransferases (E2.1.1.104) were markedly upregulated upon pathogen infection in resistant material, implying a potential role of protein methylation in conferring resistance (**Figure 6f**).

Overall, the response to rust in *B. striata* represented a complex interplay of multiple genes and pathways acting coordinately (**Figure 6g**). The response encompassed multiple pathways including oxidative stress response, reactive oxygen species scavenging, calcium ion signaling in the cell wall, energy metabolism, phytohormone signaling, and phenylpropanoid biosynthesis. Notably, phytohormone-related genes constituted the largest proportion and served as central hubs connecting various pathways through extensive gene networks.

### 3.7 qRT-PCR

A total of 10 key differentially expressed genes—*SOD1* (DN49610), *ETR* (DN531), *ERF* (DN16282), *WRKY33* (DN4172), *MYC2* (DN2177), *XTH* (DN1676), *CPK26* (DN7604), *WRKY22* (DN9292), *KCS* (DN4740), and *CALM* (DN37386)—were subjected to qRT-PCR validation analysis. The trends of transcriptome expression of the 10 genes and the qRT-PCR fluorescence quantification expression levels were basically consistent (*R* = 0.88, *p* < 0.05), which further confirmed the reliability of the transcriptome data (**Supplementary Figure S4**).

## 4 Discussion

### 4.1 Resistance performance to rust pathogen in the resistant material of *B. striata*

Orange spore clusters were clearly observed on the abaxial leaf surfaces of both materials in *B. striata* by 2 dpi. While similar sporulation patterns were initially observed in both resistant and susceptible materials, the progression of infection differed markedly. In the resistant material, both the expansion rate of infection areas and the proliferation of spore clusters were significantly slower compared to the susceptible material as the infection progressed. This pattern contrasts with the typical response in wheat to yellow (stripe) rust, where resistant cultivars typically develop necrotic lesions without sporulation (Zhao et al., 2022). The observed differences may reflect species-specific defense responses and physiological variations among rust pathotypes. The resistant phenotype observed in *B. striata* likely reflects an effective inducible resistance response rather than complete immunity, as supported by physiological and transcriptomic data. Specifically, the resistant material exhibited enhanced enzyme activity and transcriptional responsiveness, such as more DEGs during infection, broader activation of defense-related pathways, and earlier response initiation.

### 4.2 Resistance to rust pathogen by rapidly responding, activating antioxidant system and improving its growth state

Plant-induced disease resistance is well established as a temporally regulated physiological process initiated by molecular recognition events between pathogen-derived elicitors and host plant receptors (Jones and Dangl, 2006; Boutrot and Zipfel, 2017). In this study, we measured multiple physiological indices to investigate the mechanisms underlying differing rust resistance between resistant and susceptible materials of *B. striata*. Our results revealed that rust pathogen infection significantly affected the leaf chlorophyll content in both materials. This could be attributed to the orange spore stacks that were observed on the back of leaves in the different materials. Leaf chlorophyll content was affected by rust fungus parasitism, which was also observed in wheat (Sharshar et al., 2025). Interestingly, the chlorophyll response patterns to pathogen challenge were similar, and there was no significant difference between resistant and susceptible materials, suggesting that chlorophyll metabolism may not be the primary determinant of resistance differentiation in this pathosystem. Furthermore, we observed that rust pathogen inoculation triggered a rapid defense response in the resistant material, characterized by early enhancement of enzyme activity and accumulation of osmotic substances compared to susceptible plants. This rapid physiological response likely contributes to its enhanced resistance. As is well established in plant–pathogen interactions, an immediate response to pathogen infection or elicitor recognition is the oxidative burst—the rapid production of ROS (Mehdy et al., 1996; Lamb and Dixon, 1997). Here we monitored the activity of superoxide dismutase (SOD) and catalase (CAT), key enzymes involved in ROS scavenging. It showed that both enzymes exhibited a significantly faster induction within 0–2 dpi in the resistant material compared to the susceptible material. This rapid activation of antioxidant enzymes suggested that (1) the rust pathogen triggered substantial ROS production during early infection and (2) the resistant material mounted a more efficient oxidative stress response, potentially contributing to its enhanced rust resistance (Higa et al., 2001; Jan et al., 2025). Notably, the soluble sugar content peaked during the strongest defense response (2 dpi) exclusively in the resistant material, suggesting its potential dual role in rust resistance by competing with pathogens for carbon sources and maintaining cellular osmolality under stress (Benhamou, 1991; Tian et al., 2023). The resistant phenotype was further supported by a significantly higher activity of PR proteins, including chitinase and β-1,3-glucanase compared to susceptible plants (Anguelova-Merhar et al., 2001; Pei et al., 2023).

In addition, the resistant material exhibited a rapid and strong response at 2 dpi, showing a clear separation from the control in PCA analysis, followed by a return toward baseline by 8 dpi. In contrast, the susceptible material displayed only a weak initial response at 2 dpi, with its major shift occurring later at 8 dpi. This temporal divergence suggests fundamentally different defense strategies, namely, early and robust immune activation in the resistant material versus a delayed and weaker response in susceptible plants. The delayed response may be attributed to the inhibition of early immunity by effector proteins (Dodds and Rathjen, 2010).

In summary, our study revealed distinct physiological responses to pathogen infection between resistant and susceptible materials of *B. striata*. While the chlorophyll components (Chl a, Chl b, and total chlorophyll) showed parallel temporal patterns without significant genotypic differences, several key defense-related parameters exhibited marked variation. The resistant material demonstrated (1) significantly faster and stronger induction of pathogenesis-related proteins (chitinase and β-1,3-glucanase), (2) elevated proline and soluble protein levels during early infection stages, and (3) unique dynamic patterns in antioxidant enzymes (SOD and CAT) and phenylpropanoid pathway activity (PAL). Notably, the transient surge in soluble sugar content observed specifically in resistant plants at 2 dpi suggests its potential role as a rapid-response metabolite in rust defense. The soluble sugar may serve as a carbon source for energy-intensive defense responses, act as signaling molecules to amplify immune responses (Bolouri Moghaddam and Van den Ende, 2012), or coordinate with other defenses. All of the results above indicated that the resistant material of *B. striata* exhibited a more rapid and robust response to rust pathogen infection compared to susceptible plants, characterized by multi-layered defense response, encompassing osmotic regulation, secondary metabolism, and PR protein activation, collectively contributing to its effective resistance mechanism against rust infection.

### 4.3 Key genes involved in the resistance to rust pathogen

The transcriptomic analysis revealed significantly more DEGs and activated pathways in rust-resistant *B. striata* compared to susceptible plants following pathogen inoculation (**Supplementary Figures S1**, **S4**). Notably, the defense response peaked at different time points between materials—2 dpi for resistant plants and 8 dpi for susceptible plants, suggesting distinct temporal patterns of immune activation. The susceptible material’s delayed defense activation, with ROS, calcium signaling, and *WRKY33*-mediated responses only reaching significant levels at 8 dpi, reflect both reduced pathogen sensitivity and compromised early warning systems (Dodds and Rathjen, 2010). The delayed response probably caused by impaired pathogen recognition disrupted early signaling of hormonal cross-talk or inefficient resource mobilization for defense. This was consistent with the physiological results of PAL, soluble sugar, soluble protein, SOD, and so on, implying its being insensitive to rust pathogen results and excessive resource expenditure on futile late-stage defenses. The transcriptomic results below further demonstrated that the resistant material of *B. striata* exhibited both faster and stronger responses to rust pathogen infection.

The KEGG pathway analysis revealed a significantly greater activation of secondary metabolite-related pathways in resistant plants compared to susceptible plants at 2 dpi. Notably, phenylpropanoid biosynthesis was exclusively enriched in the H2 vs. HCK comparison group. The phenylpropanoid pathway plays a well-established role in plant defense, generating diverse metabolites including lignin and flavonoid precursors. These compounds contribute to resistance through multiple mechanisms, such as reinforcing physical barriers via lignin deposition, producing antimicrobial flavonoids, and restricting pathogen hyphae to epidermal cell layers, thereby preventing systemic colonization (Yao et al., 2021; Shang et al., 2022). Our transcriptomic analysis revealed a significant downregulation of key lignin biosynthesis genes (*PAL*, *4CL*, *COMT*, *CYP98A*, E1.11.17, *CAD* and *REF1*) in the resistant material of *B. striata* compared to susceptible plants. This suppression of lignin biosynthesis may redirect carbon flux within the phenylpropanoid pathway, potentially enhancing the production of other defensive secondary metabolites (Baxter and Stewart, 2013). Supporting this hypothesis, we observed a significant enrichment of flavonoid-related pathways (“flavonoid biosynthesis”, “flavone and flavonol biosynthesis”) and “biosynthesis of various plant secondary metabolites” in the H2vsHCK comparison group. Notably, genes involved in scopolin biosynthesis showed a marked upregulation in resistant plants. This finding is particularly significant as scopolin has recently been identified as a key phytoprotective compound contributing to disease resistance (Song and Wu, 2024). We therefore proposed that the enhanced rust resistance in *B. striata* may result from metabolic reprogramming toward alternative defensive compounds (phenolics, terpenoids) rather than lignin production (Tsitsekian et al., 2021), a strategy observed in other pathosystems involving trade-offs between structural and chemical defenses (Carmona et al., 2011).

Extensive studies have established that phytohormones serve as central regulators orchestrating plant immune responses by coordinating downstream defense gene networks (Yang et al., 2017; Li et al., 2022; Zou et al., 2024). Our temporal transcriptome analysis revealed striking differences in hormonal signaling between resistant and susceptible materials of *B. striata.* The differing temporal regulation and magnitude of hormonal responses between the resistant and susceptible materials of *B. striata* likely constitute a key determinant of their contrasting rust resistance. In this study, the transcriptomic analysis revealed distinct temporal patterns in “plant hormone signal transduction” pathway activation—peaking at 2 dpi in the resistant material of *B. striata* while at 8 dpi in susceptible plants. Besides that, our analysis revealed fundamental differences in hormonal reprogramming between resistant and susceptible plants at 2 dpi. In susceptible plants, auxin signaling was prominently activated through the upregulation of both *IAA* repressors and *ARF* transcriptional activators, while the resistant plants showed a strong downregulation of *SAUR* growth effector. This diametric regulation suggests that auxin signaling bifurcates defense strategies, promoting susceptibility (via IAA/ARF activation) or resistance (via *SAUR* suppression). Concurrently, resistant plants exhibited coordinated defense hormone activation through JA pathway induction (*JAZ* repressor downregulation), ABA signaling activation (*ABF* upregulation and *PP2C* downregulation), and SA response potentiation (*TGA* factor upregulation). In contrast, the susceptible plants showed BR signaling suppression (*BIN2* and *BKI1* upregulation), acting as a defense-growth integrator, and growth-promoting pathway activation (*GID2* upregulation). The observed difference suggested that resistant plants employed early hormone crosstalk (JA-ABA-BR) to release the JAZ-mediated repression of defense genes, activate *ABF*-independent stress responses, and optimize resource allocation via *GID2* downregulation. However, the susceptible plants exhibited delayed and dysregulated signaling, such as prolonged *JAZ* repression to limit JA responses, *PP2C*-mediated ABA desensitization, and BR-GA interference through *BIN2*/*DELLA*. These findings align with emerging models of hormonal immunity JA-ABA synergy enhancing rust resistance (Zou et al., 2024), BR signaling fine-tunes defense–growth tradeoffs (Ren et al., 2024), and auxin suppression promoting SA-dependent defenses (Navarro et al., 2006). These results demonstrated that resistant plants prioritized defense through the early suppression of growth-related signals (auxin, cytokinin, and GA), concurrent activation of defense hormones (SA, JA, and ABA), and strategic BR pathway modulation. This precise hormonal re-balancing initiates growth–defense trade-offs earlier in resistant plants, enabling faster immune responses compared to susceptible plants.

The transcriptomic and functional analyses revealed a targeted reprogramming of cell wall metabolism in resistant plants during early rust infection (2 dpi). The key biosynthetic genes of structural component synthesis were significantly downregulated (*CESA*, *GAUT*, *CSLA*, and *COMT*), but the key genes of callose synthesis (*VPS50* and *CALS*), namely, defense-specific pathways, were upregulated in the high-resistance material. Therefore, the resistant material of *B. striata* likely adopted a cell wall remodeling strategy that appeared optimized for rapid defense rather than structural reinforcement—diverting resources from conventional wall components toward callose deposition and fungal wall interference. This aligned with recent models where callose microdomains served as physical and chemical defense platforms (Pring et al., 2023), while simultaneous chitin metabolism disruption synergistically enhances resistance (Saberi Riseh et al., 2024).

### 4.4 Mechanism of resistance to rust pathogen in *B. striata*

**Figure 7:**
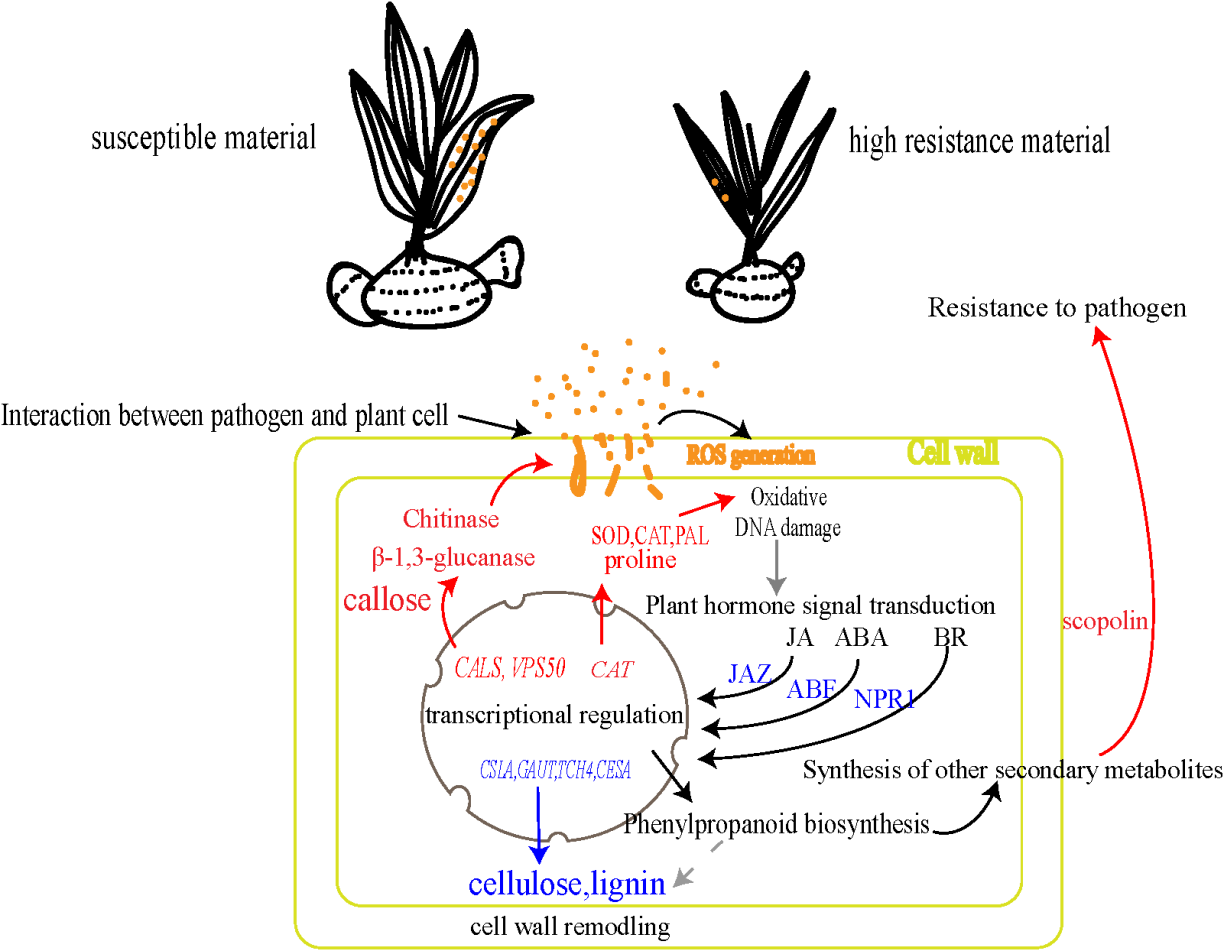
Mechanism of resistance to rust pathogen in *B. striata*. The blue arrows/text indicate decreased content or downregulated genes, while the red arrows/text denote increased content or upregulated genes.

In summary, the rust defense response in *B. striata* represented a complex interplay of multiple physiological and molecular pathways. Based on our findings, we proposed a mechanistic model (**Figure 7**). Early recognition and signaling: ROS burst was triggered immediately upon pathogen detection at the cell wall. Phytohormone signaling cascades (JA, ABA, and BR) amplified and transduced the defense signals. Transcriptional reprogramming: rapid activation of defense-related gene networks and coordinated expression of protective enzymes and metabolites. Multi-layered defense execution: antioxidant system (enhanced activity of CAT, SOD, and PAL enzymes coupled with proline accumulation), physical barriers (dynamic cell wall remodeling through callose deposition and regulated activity of cellulases/pectinases), and direct antimicrobial action: (production of chitinase, β-1,3-glucanase, and phytoalexin scopolin). This comprehensive response effectively restricts pathogen spread through the simultaneous deployment of physical barriers, chemical defenses, and signaling network modulation, contrasting sharply with the delayed and weaker response in susceptible plants. This delayed response could be contributed to the immune suppression by rust pathogen effectors in the susceptible plants, just as shown in other rust fungi (Ramachandran et al., 2017).

## 5 Conclusion

Our findings demonstrated that rust resistance in *B. striata* involved a coordinated multi-layered defense system comprising early physiological response, rapid activation of defense enzymes, significant accumulation of osmolytes, and faster and stronger activation of transcriptional reprogramming in resistant plants. Here we elucidated the comprehensive defense architecture underlying rust resistance in *B. striata*, identified crucial regulatory hubs in phytohormone signaling (JA/ABA/BR), antioxidant systems and phenylpropanoid metabolism, and revealed potential genetic targets (including PAL, β-1,3-glucanase, callose, and scopolin biosynthesis genes) for precision breeding strategies. The characterized defense modules provide a molecular toolkit to develop durable resistance in *Bletilla* species through either marker-assisted selection or transgenic approaches.

## Data Availability

The datasets presented in this study can be found in online repositories. The names of the repository/repositories and accession number(s) can be found in the article/**Supplementary Material**.
