# Supplementary material for: Transcriptomics combined with physiological analysis provides insights into the mechanism of resistance to Coleosporium bletiae in Bletilla striata
Source: Front Plant Sci. 2025 Jul 14;16:1604512. doi: 10.3389/fpls.2025.1604512 (PMC12301390; doi:10.3389/fpls.2025.1604512)
Supplement: Supplementary file 1 [file DataSheet1.pdf]

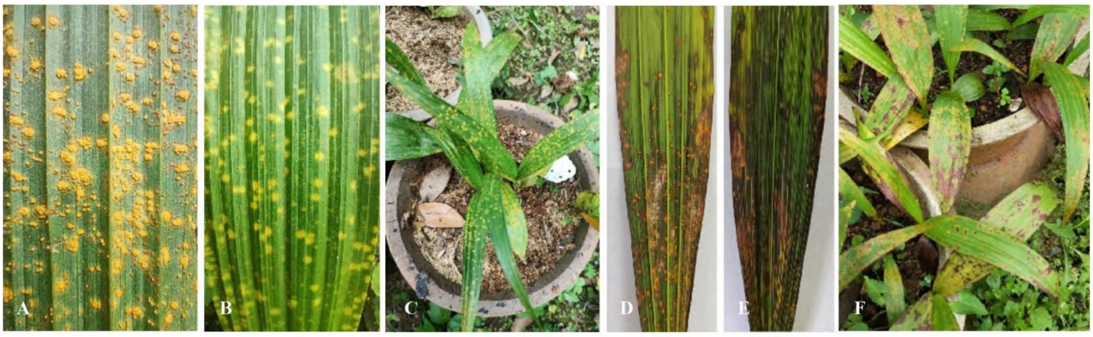

Figure S1 Symptoms of rust on B. striata; A, B. leaves infected with urediospore (A. back; B. front); C. plants infected with urediospore; D, E. leaves infected with teleutospore (D. back; E. front); F. plants infected with teleutospore.

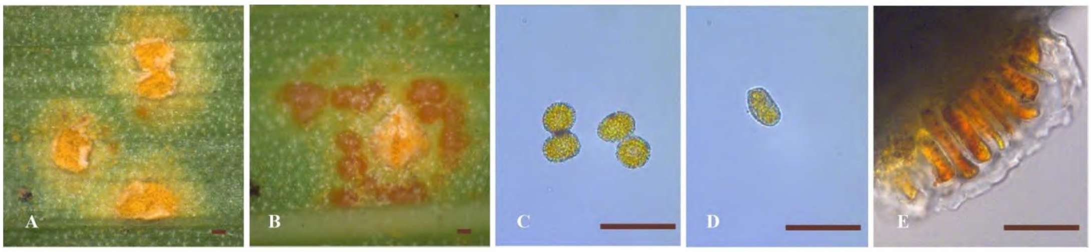

Figure S2 Morphological characteristics of rust pathogen; A,B. uredinium; C,D. urediospore; E. teleutospore; scale bar: A,B=0.1mm; C-E=50um.

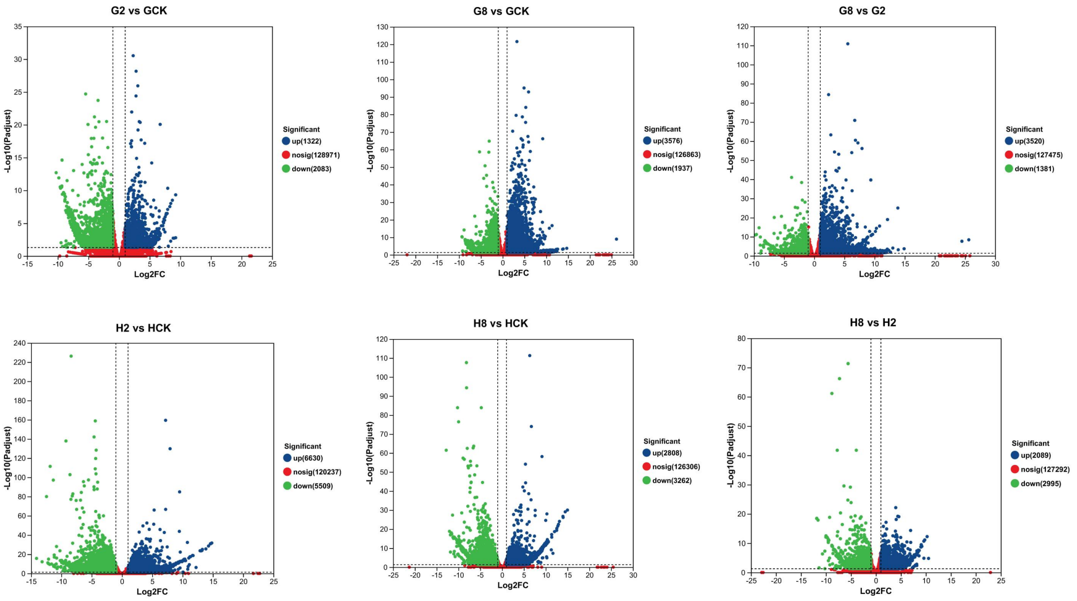

Figure S3. Number of differentially expressed genes (DEGs) between different groups.GCK, G2 and G8 represent samples on 0, 2 and 8 dpi in susceptible material; HCK, H2 and H8 represent samples on 0, 2 and 8 dpi in resistant material.

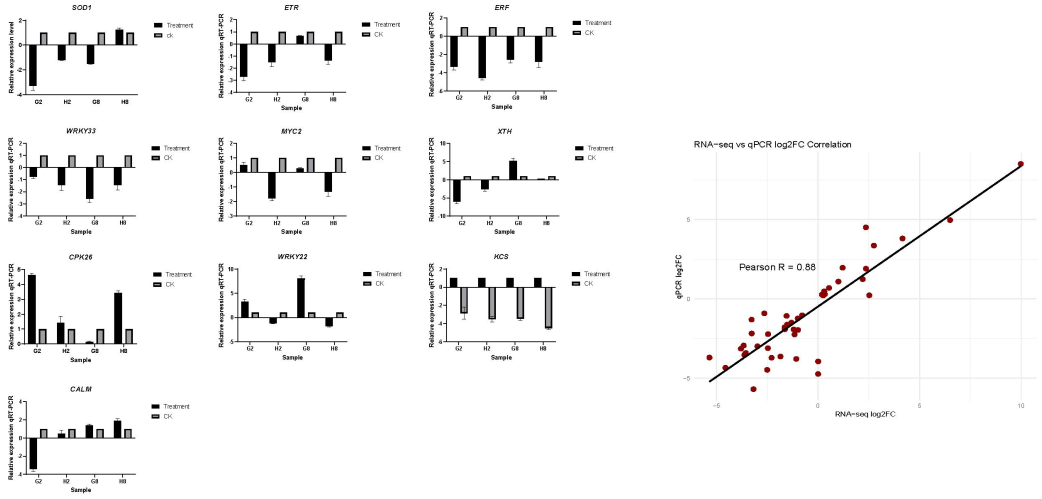

Figure S4. Validation of RNA-seq results by qPCR; a, qPCR results of 10 candidate genes; b, correlation of log_2fold-changes for 10 candidate genes between RNA-seq (TPM normalized) and qPCR. The strong positive correlation (r= 0.88, p <0.01) confirms the reliability of transcriptome data.
